# Novel Insights into TSC22D Family Genes in Metabolic Diseases and Cancer

**DOI:** 10.3390/biom16010179

**Published:** 2026-01-22

**Authors:** Wen Shen, Cong Shen, Yang Jiao, Xia Deng, Jue Jia, Guoyue Yuan

**Affiliations:** Department of Endocrinology and Metabolism, The Affiliated Hospital of Jiangsu University, Institute of Endocrine and Metabolic Diseases, Jiangsu University, Zhenjiang 212001, China; 2112313256@stmail.ujs.edu.cn (W.S.); 2212313021@stmail.ujs.edu.cn (C.S.); 2222313027@stmail.ujs.edu.cn (Y.J.); 1000012396@ujs.edu.cn (X.D.)

**Keywords:** TSC22D family gene, biological function, glucose and lipid metabolism, cancer

## Abstract

Transforming growth factor-beta 1 (TGF-β1)-stimulated clone 22 domain (TSC22D) family genes (including *TSC22D1-TSC22D4*) were identified as transcription factors. It has been demonstrated that they display multiple functions due to proteins’ isoforms, redundancy, and other factors. Formerly, researchers mainly focused on its functions, like controlling cell growth and development, cell apoptosis, and balance of osmotic pressure in vivo. Nowadays, growing evidence indicates that they also play an important role in metabolic regulation and the immune system and are expected to be a new potential target for the treatment of diabetes or obesity. Despite this, it has been shown that TSC22D family genes have an inhibitory effect in multiple tumors. In this review, we significantly synthesized advances in metabolism, showing that *TSC22D3* could control lipid accumulation via modulating adipogenesis and adipose differentiation, while *TSC22D4* could regulate insulin sensitivity and gluconeogenesis by affecting Akt (serine/threonine kinase, also known as protein kinase B, or PKB) phosphorylation. Moreover, we provide novel insights, including the fact that TSC22D family genes function as a double-edged sword in cancer due to the type of tumor and tumor microenvironment (TME).

## 1. Introduction

TSC22D family genes represent a critical group of transcriptional regulators that have garnered increasing interest in biomedical research due to their multifaceted roles in cellular physiology. These genes encode proteins characterized by a conserved leucine zipper domain, which facilitates protein–protein interactions essential for their function as transcriptional modulators [1,2]. Historically, members of the TSC22D family have been implicated in fundamental cellular processes such as stress response, proliferation, differentiation, and apoptosis [3]. Their ability to integrate various intracellular and extracellular signals positions them as pivotal modulators of cell fate decisions. The complexity of their regulatory networks underscores the importance of this gene family in maintaining cellular homeostasis and adapting to environmental challenges.

In recent years, the focus on TSC22D family genes has expanded beyond basic cell biology to encompass their involvement in complex diseases, particularly metabolic disorders and cancer. Metabolic diseases, including diabetes mellitus, non-alcoholic fatty liver disease (NAFLD), and obesity, represent a significant and escalating global health burden. Emerging evidence suggests that TSC22D family genes contribute to the pathogenesis of these metabolic diseases by modulating key signaling pathways that regulate glucose and lipid metabolism, insulin sensitivity, and inflammatory responses [4,5]. This evidence has paved the way for a fresh perspective on the molecular foundations of metabolic disorders, highlighting the regulatory framework in which TSC22D proteins play a crucial role.

Concurrently, cancer remains one of the leading causes of morbidity and mortality worldwide, characterized by uncontrolled cell proliferation, evasion of apoptosis, metastatic potential, and, often, resistance to conventional therapies. TSC22D family genes have been increasingly recognized for their dualistic roles in tumorigenesis. Studies have demonstrated that these genes can function either as oncogenes or tumor suppressors depending on the cellular context and cancer type [6,7,8]. Their involvement in regulating tumor cell proliferation, migration, invasion, and chemoresistance highlights their complexity and therapeutic relevance. Understanding the molecular mechanisms by which TSC22D genes influence tumor biology may facilitate the development of targeted therapies, improve prognostic assessments, and overcome treatment resistance.

This review seeks to present a thorough overview of the existing knowledge concerning TSC22D family genes’ structure and functional characteristics, highlighting their emerging significance in metabolic disorders and cancer. We will critically analyze recent studies that clarify how TSC22D proteins regulate transcriptional mechanisms influencing disease-related cellular activities. Additionally, we will investigate the potential of targeting TSC22D family genes as biomarkers or therapeutic options while considering the obstacles and future trajectories in this developing area. Through this systematic exploration, we aim to emphasize the importance of TSC22D family genes in disease mechanisms and highlight their potential as innovative molecular targets in clinical settings.

## 2. The Role of the TGF-β Signaling Pathway in the Crosstalk Between Development and Metabolism

The TGF-β signaling pathway, as an evolutionarily highly conserved cellular communication system, plays a central role in developmental biology and metabolic regulation. Research indicates that TGF-β superfamily genes are widely distributed across the animal kingdom, participating in the regulation of diverse cellular processes and maintaining overall health homeostasis [9]. During vertebrate development, members of the TGF-β family, including subfamilies such as TGF-β and bone morphogenetic protein (BMP), guide embryonic morphogenesis and organogenesis by precisely regulating cell proliferation, differentiation, and apoptosis [9,10]. The classic Smad-dependent pathway of the TGF-β signaling is a key molecular mechanism regulating cell fate. This pathway initiates when TGF-β ligands (including TGF-β1, β2, and β3) bind to type II receptors (TβRII) on the cell membrane, subsequently recruiting and phosphorylating type I receptors to form an active receptor complex [11]. Meanwhile, TGF-β receptors can directly or indirectly activate multiple non-Smad signaling pathways, including MAPK (such as ERK, JNK, and p38), the PI3K/Akt/mTOR pathway, and the Rho GTPase family [12,13]. In recent years, with the advancement of systems biology and metabolomics, the central role of TGF-β signaling in systemic and tissue-specific metabolic regulation has become increasingly prominent. In hepatic metabolism, TGF-β signaling influenced the expression of genes related to glucose and lipid metabolism by regulating the stability of SMAD proteins [14]. In adipose tissue, TGF-β/Smad3 signaling activation was closely associated with adipose fibrosis while simultaneously inhibiting beige adipocyte differentiation [15]. Dysfunction of TGF-β signaling is closely associated with various human metabolic disorders. In diabetic nephropathy, TGF-β signaling regulated extracellular matrix metabolism through both SMAD-dependent and non-canonical pathways, thereby promoting renal fibrosis [16]. In skeletal metabolism, members of the TGF-β superfamily, such as BMPs, influenced bone homeostasis by regulating osteoblast differentiation and bone development [17]. These findings highlight the importance of the TGF-β pathway in integrating developmental and metabolic signals.

## 3. The Structure, Function, and Regulation of TSC22D Family Genes

TSC22D family genes contain three distinct domains: an N terminus TSC box, a middle leucine zipper (LZ) domain, and a C terminus proline-rich domain [18]. They were originally identified because of their ability to bind to other transcription factors and participate in the regulation of cell proliferation and differentiation [1]. The proteins encoded by TSC22D family genes are expressed in many different tissues, such as the brain, salivary gland, colon, kidney, ovary, testis, and so on [19].

**Figure 1:**
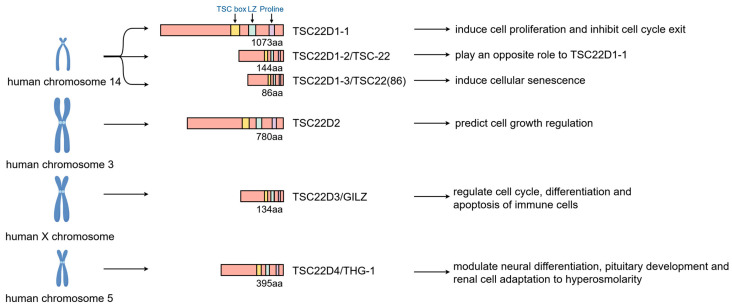
The location, structure, and functions of TSC22D family genes. TSC22D family genes contain three distinct domains: an N terminus TSC box, a middle leucine zipper (LZ) domain, and a C terminus proline-rich domain. *TSC22D1-TSC22D4* genes are respectively located on human chromosomes 14, 3, X, and 5. They encode different proteins to perform various functions. (Created in Figdraw. Wen Shen. (2026) https://www.figdraw.com).

The *TSC22D1* gene, formerly named TGF-β-stimulated clone-22 (TSC-22), is located on human chromosome 14 and encodes proteins including *TSC22D1-1*, *TSC22D1-2* (*TSC-22*), and *TSC22D1-3* (*TSC22*), which are, respectively, 1073, 144, and 86 amino acids (aa) long [1,2] (Figure 1). These isoforms are produced by selective splicing. Significantly, compared with *TSC22D1-1*, *TSC22D1-2* has a much shorter N-terminal region and plays the opposite role [1]. Various growth factors or hormones, as well as cellular stress, have been reported to increase the expression of TSC22D1-2 protein, which in turn induces cell differentiation and apoptosis of several cells when overexpressed and inhibits cell proliferation [20,21,22,23,24,25,26,27]. Furthermore, TSC22D1-2 is essential for embryonic development [25]. Apart from this, DNA damage could up-regulate TSC22D1-3, a protein that induces cellular senescence and thus plays an anti-tumor role [2,28].

*TSC22D2* lies in human chromosome 3 and encodes proteins composed of 780 aa [29]. It is a predictor of cell growth regulation and is involved in the activity of intestinal progenitor cells [30,31]. *TSC22D3* is situated on the human X chromosome and mainly encodes glucocorticoid-induced leucine zipper (GILZ), a protein consisting of 134 aa, which shares 78% homology with *TSC22D1-2* in coding portion [32]. TSC22D3 was initially recognized as an endogenous protein [33]. As a spermatogenesis regulator [34], it was also reported to participate in controlling the cell cycle, differentiation, and apoptosis of immune cells [35]. Studies now show that TSC22D3 is rapidly and inevitably induced by glucocorticoids (GCs), mediating the anti-inflammatory, immunosuppressive, and antiproliferative effects of GCs in many cell types, including regulatory T-cells [36,37,38].

*TSC22D4* is located on chromosome 5 and can encode the human TSC-22 homologous gene-1 (THG-1) protein composed of 395 aa, which shares 75% sequence homology with *TSC22D1-2* and 67% homology with *TSC22D3* [32]. It was initially cloned on the basis of sequence homology to growth factor- and glucocorticoid-induced TSC22 transcription factor family members D1 and D3 and was subsequently discovered to have transcriptional activity when fused to a heterologous DNA-binding domain [39]. TSC22D4 could modulate neural differentiation [40], pituitary development [41], and renal cell adaptation to hyperosmolarity [42]. TSC22D family proteins could also interact with each other. Recent evidence suggests that the TSC22D4-TSC22D1-2 heterodimer is involved in cell proliferation escape and cell cycle exit, whereas TSC22D1-1 promotes cell proliferation and inhibits cell cycle exit [1].

The genes belonging to this family are remarkably preserved and encode proteins whose roles are challenging to discern due to the presence of isoforms, redundancy, and other factors [43]. Despite this, extensive studies have shown that they have an inhibitory effect in multiple tumors. Recently, many scholars have found that they also play an important role in metabolic regulation and the immune system and are expected to be a new target for the treatment of diabetes or obesity [44,45,46,47,48].

## 4. The Role of TSC22D Family Genes in Lipid Metabolism

Lipid homeostasis is fundamental to maintaining energy supply, cell membrane synthesis, and signal transduction in mammals. Its disruption can trigger a series of metabolic disorders, including fatty liver disease, hypertriglyceridemia, and diabetes. The TSC22D family, as an emerging family of transcription regulators, modulates key pathways in lipid metabolism. Members of this family participate in regulating systemic lipid distribution and energy balance by modulating hepatic VLDL secretion, lipid synthesis, mitochondrial function, and the insulin-Akt signaling axis. Dysfunction within this family is closely associated with metabolic disorders such as cancer cachexia, NAFLD, and insulin resistance.

### 4.1. TSC22D1 and Lipid Metabolism

**Figure 2:**
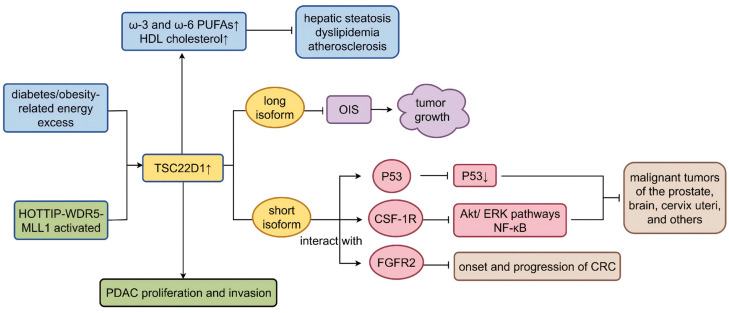
Roles of TSC22D1 in lipid metabolism and cancer. PUFAs: polyunsaturated fatty acids; HDL: high-density lipoprotein; HOTTIP-WDR5-MLL1 HOXA: transcript at the distal tip-WD repeat domain 5-mixed lineage leukemia 1; PDAC: pancreatic ductal adenocarcinoma; OIS: oncogene-induced senescent; CSF-1R: colony stimulator factor 1 receptor; FGFR2: fibroblast growth factor receptor 2; CRC: colorectal cancer; Akt: serine/threonine kinase; ERK: extracellular signal-regulated kinase; NF-κB: nuclear factor kappa-B. ↑ represents up-regulated ↓ represents down-regulated, and → represents promoting ⊣ represents inhibiting. (Created in Figdraw. Wen Shen. (2026) https://www.figdraw.com).

In terms of metabolism, Uchida D et al. found that transgene-mediated overexpression of TSC-22 led to marked obesity in mice, and most obese transgenic mice exhibited significant hepatocellular steatosis, while elevated blood glucose and renal fibrosis were not observed [25]. TSC22D1 was later recognized as a key protein involved in the synthesis and catabolism of fatty acids. For example, it could promote the biosynthesis of ω-3 and ω-6 polyunsaturated fatty acids (PUFAs). Additionally, its dysregulation could result in hepatic steatosis [49]. Consistently, previous studies demonstrated that TSC22D1 controlled systemic high-density lipoprotein (HDL) cholesterol metabolism through transcriptional regulation of the HDL cholesterol loading. Its overexpression could increase the HDL cholesterol level. Hepatic TSC22D1 expression was governed by systemic energy status, and hepatic *TSC22D1* mRNA, and protein levels were up-regulated under conditions of energy excess [50]. It can be seen that elevated hepatic TSC22D1 levels in the context of diabetes/obesity-related energy excess are feedback of self-stress designed to antagonize dyslipidemia and atherosclerosis (Figure 2). Since Uchida D. et al. did not examine dietary intake, excretion, and motor behavior in the metabolic gauge during the experiments and their mice were transgenic, we reckon that high expression of TSC22D1 benefits obesity.

### 4.2. TSC22D2 and Lipid Metabolism

It is well known that abdominal obesity is usually measured by waist circumference (WC) and waist-to-hip ratio (WHR), while general obesity is measured by body mass index (BMI). A study found that a single nucleotide polymorphism (SNP) (rs1868673) near the *TSC22D2* gene was associated with WC without adjustment for BMI in an East Asian population, and the relationship between this locus and WC was attenuated after adjustment for BMI [51]. It was also reported that the locus might be related to circulating adiponectin levels [52]. Adiponectin is a plasma protein principally secreted by adipocytes, and its level is negatively correlated with the risk of insulin resistance index, type 2 diabetes mellitus (T2DM), and atherosclerosis [53,54,55,56,57]. These results suggest that TSC22D2 may be involved in abdominal obesity and offer guidance for future studies on the effects of TSC22D2 on lipid metabolism.

### 4.3. TSC22D3 and Lipid Metabolism

Notably, GCs modulate adipose metabolism and endocrine function and have a major impact on full differentiation of adipocyte precursors and maintenance of adipogenic gene expression [37]. Although we are clear about the function of TSC22D3 in immune cells, the role of TSC22D3 in adipocytes has not been addressed yet. Therefore, it was interesting to see whether TSC22D3, a major target of GC, was also involved in the regulation of adipokines. TSC22D3 can be expressed in both cultured human adipose tissue and newly differentiated human adipocytes, and it is up-regulated by dexamethasone (Dex) via glucocorticoid receptor (GR) [58,59]. Obesity is known to induce chronic inflammation, which is characterized by a marked increase in the concentration of pro-inflammatory factors tumor necrosis factor-α (TNF-α) and interleukin-6 (IL-6) in the blood [4]. It was reported that TNF-α decreased the expression of TSC22D3 in human adipocytes, and TSC22D3 in turn was a negative regulator of pro-inflammatory signaling pathways. Expanding on that, TSC22D3 attenuated the pro-inflammatory effects of TNF-α in human adipocytes by limiting the nuclear translocation of nuclear factor kappa-B (NF-κB). Since most anti-inflammatory effects of GCs depend on crosstalk with the mitogen-activated protein kinase (MAPK) signaling pathway, the inspired researchers demonstrated that TSC22D3 interacted with activated Ras to suppress phosphoinositide 3-kinase (PI3K)/Akt and MAPK/extracellular signal-regulated kinase (ERK) pathways, thereby inhibiting cell growth and differentiation [38]. In addition, TSC22D3 could regulate B cells’ activity and the development of inflammatory diseases by repressing the function of activator protein-1 (AP-1) [60]. AP-1 is a key transcription factor for immune cell activation during inflammation [61]. TSC22D3 could also bind to SMAD family member 2 (SMAD2), which leads to the phosphorylation of SMAD2 and thus to the optimal induction of forkhead box protein P3 (FoxP3) and regulatory T cell (Tregs) proliferation [62].

TSC22D3 was found to affect adipokines such as IL-6 and leptin, both of which had pro-inflammatory effects [37]: *TSC22D3* knockdown up-regulated leptin and IL-6 expression and increased GCs’ stimulation of leptin secretion, while it had no effect on the GCs’ inhibition of IL-6. Furthermore, *TSC22D3* silencing reduced the mRNA levels of lipocalin, an anti-inflammatory and insulin-sensitive adipokine, but did not make a significant impact on total 24 h lipocalin secretion, which might be related to its high cellular stores. It did not influence adipose triglyceride lipase (ATGL) protein levels either. ATGL is used as a marker of adipocyte differentiation. Consistently, *TSC22D3* overexpression downregulated leptin and IL-6 expression and did not markedly affect lipocalin mRNA levels and secretion over 24 h [63]. Therefore, TSC22D3 may affect systemic energy homeostasis and metabolism through the downregulation of adipocyte leptin and IL-6 production and enhancement of lipocalin expression.

**Figure 3:**
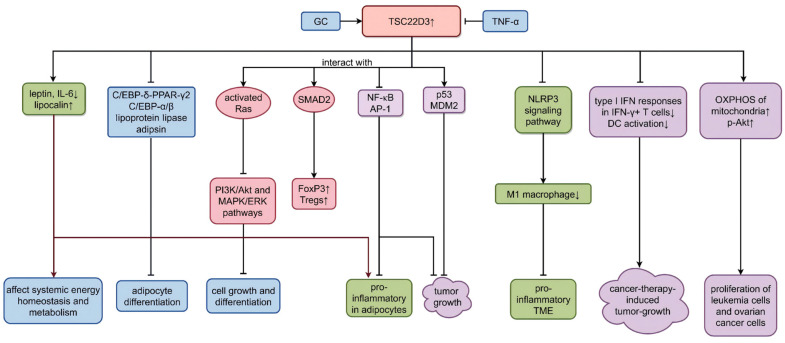
Roles of TSC22D3 in lipid metabolism and cancer. GC: glucocorticoid; TNF-α: tumor necrosis factor-α; IL-6: interleukin-6; C/EBP-δ: CCAAT/enhancer-binding protein-δ; PPAR-γ2: peroxisome-proliferator-activated receptor-γ2; PI3K/Akt: phosphoinositide 3-kinase/serine/threonine kinase; MAPK/ERK: mitogen-activated protein kinase/extracellular signal-regulated kinase; SMAD2: SMAD family member 2; FoxP3: forkhead box protein P3; Tregs: regulatory T cells; NF-κB: nuclear factor kappa-B; AP-1: activator protein-1; MDM2: murine double minute 2; NLRP3: NOD-like receptor protein 3; IFN: interferon; DC: dendritic cell; OXPHOS: oxidative phosphorylation. ↑ represents up-regulated ↓ represents down-regulated, and → represents promoting ⊣ represents inhibiting. (Created in Figdraw. Wen Shen. (2026) https://www.figdraw.com).

Previous studies showed that GCs also induced differentiation of human bone marrow mesenchymal stem cells to adipocytes. It was a cascade reaction in which GC induced the expression of CCAAT/enhancer-binding protein-δ (C/EBP-δ), followed by direct binding of C/EBP-δ to the peroxisome-proliferator-activated receptor-γ2 (PPAR-γ2) promoter, which is a key regulator of adipogenesis. This activated the expression of PPAR-γ2, after which PPAR-γ2 initiated the adipocyte differentiation program [64]. TSC22D3 was later identified as a sequence-specific DNA-binding factor. It bound to C/EBP tandem DNA elements in the PPAR-γ2 promoter and repressed C/EBP-δ-mediated transcription. The C/EBP transcription factor family consists of three members, C/EBP-α, C/EBP-β, and C/EBP-δ, all of which are involved in the induction of adipocyte differentiation. When bound to DNA, these factors can form homodimers or heterodimers with other family members. In short, both C/EBP-δ and TSC22D3 were transcriptionally activated by GCs, but C/EBP-δ induced PPAR-γ2 expression, while TSC22D3 inhibited it. In addition, TSC22D3 suppressed other key adipogenic regulators (e.g., C/EBP-α) and downstream adipocyte differentiation marker genes (e.g., lipoprotein lipase and adipsin) to block adipocyte differentiation in 3T3-L1 cells [18,65] (Figure 3).

### 4.4. TSC22D4 and Lipid Metabolism

**Figure 4:**
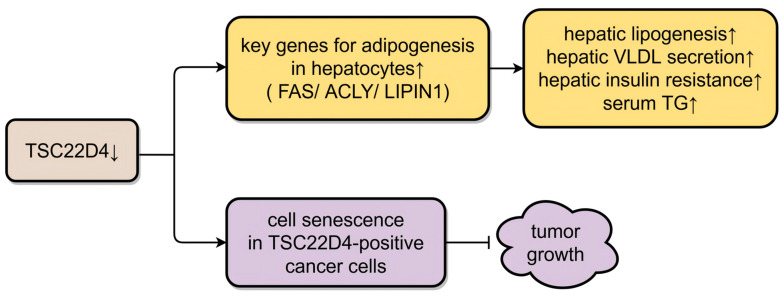
Roles of TSC22D4 in lipid metabolism and cancer. FAS: fatty acid synthase; ACLY: ATP citrate lyase; LIPIN1: lipid phosphate phosphohydrolase 1; VLDL: very-low-density lipoprotein; TG: triglyceride. ↑ represents up-regulated ↓ represents down-regulated, and → represents promoting ⊣ represents inhibiting. (Created in Figdraw. Wen Shen. (2026) https://www.figdraw.com).

Furthermore, the study demonstrated that TSC22D4 also controlled hepatic and systemic lipid metabolism in healthy wild-type mice and mice fed a high-fat diet [66]. The results of liver-specific *TSC22D4* loss- and gain-of-function experiments remained consistent in each of these two mouse types. Deletion of hepatic *TSC22D4* significantly decreased hepatic triglyceride (TG) levels and increased serum levels of total TG and very-low-density lipoprotein (VLDL)-associated TG. High levels of TSC22D4, under both normal and hypercaloric feeding conditions, evoked serum TG consumption and hypo-beta-lipoproteinemia as well as hepatic lipid accumulation. Other metabolic parameters, such as body weight, liver weight, and serum and hepatic cholesterol levels, were not significantly different from those of the control group. The underlying mechanism might be that the knockdown of *TSC22D4* in mouse and human hepatocytes resulted in the expression of key genes for adipogenesis, including fatty acid synthase (FAS), ATP citrate lyase (ACLY), and lipid phosphate phosphohydrolase 1 (LIPIN1), a gene engaged in hepatic VLDL generation. LIPIN is a cytoplasmic phosphatidic acid phosphatase. It was reported to enhance hepatic lipogenesis, hepatic VLDL secretion, and hepatic insulin resistance [66] (Figure 4). These results suggest that TSC22D4 regulates serum TG homeostasis by controlling hepatic VLDL release. Overall, hepatic TSC22D4 may mediate peripheral energy deprivation in metabolic wasting diseases.

Given that patients with progressive NAFLD commonly possess insulin resistance and aberrant hepatic lipid accumulation and that the progression of NAFLD ranges from simple steatosis to non-alcoholic steatohepatitis (NASH) to fibrosis, researchers explored the role of TSC22D4 in mouse models of NASH and liver fibrosis and found that knockdown of *TSC22D4* in hepatocytes reduced hepatic lipid accumulation [67].

## 5. The Role of TSC22D Family Genes in Glucose Metabolism

**Figure 5:**
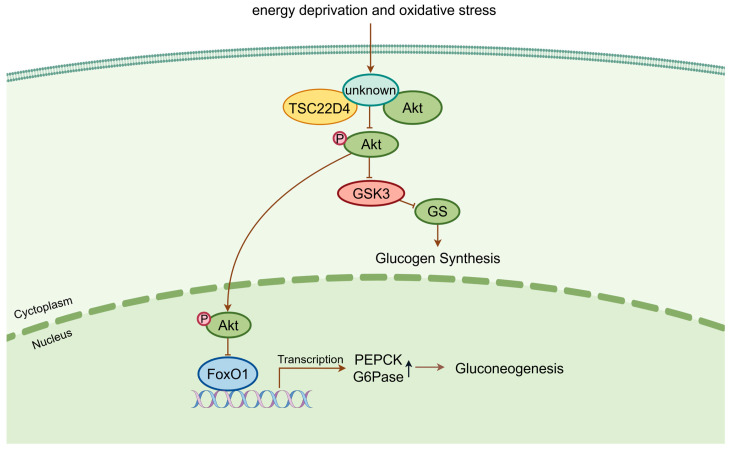
Schematic representation of the mechanism of TSC22D4 in glucose metabolism. Akt: serine/threonine kinase; GSK3: glycogen synthase kinase-3; GS: glycogen synthase; FoxO1: forkhead box O1; PEPCK: phosphoenolpyruvate carboxykinase; G6Pase: glucose-6-phosphatase. ↑ represents up-regulated ↓ represents down-regulated, and → represents promoting ⊣ represents inhibiting. (Created in Figdraw. Wen Shen. (2026) https://www.figdraw.com).

There have been few reports about TSC22D family genes and glucose metabolism. Now, new research demonstrates that *TSC22D1* depletion in INS-1E cells enhances the expression of key beta cell identity genes, including insulin 1 (Ins1), insulin 2 (Ins2), pancreatic and duodenal homeobox 1 (Pdx1), and others and promotes glucose-stimulated insulin secretion without altering the intracellular insulin content [68]. In another recent study, TSC22D4 was identified as a key driver of insulin resistance and poor glucose tolerance in obesity and T2DM. Inhibition of hepatic TSC22D4 not only improved systemic insulin sensitivity and glucose tolerance without inducing hypoglycemic episodes in healthy conditions but also modulated insulin sensitivity under conditions of metabolic dysfunction induced by a high-fat diet [5]. The main mechanism was that the TSC22D4-Akt interaction controlled Akt phosphorylation [69]. Loss of *TSC22D4* significantly increased the phosphorylation of Akt/PKB and its downstream targets [5], thereby enhancing insulin sensitivity. Furthermore, the interaction of TSC22D4 with Akt reduced the gluconeogenic potential and was sensitive to nutritional state—it was strong under starvation and weakened by high insulin/glucose levels, suggesting that TSC22D4 is a metabolic sensor in insulin signaling pathways [44]. In summary, selective knockdown of TSC22D4 in type 2 diabetic mouse hepatocytes could ameliorate glucose metabolism by reducing circulating glucose concentrations, increasing hepatic insulin sensitivity, and enhancing Akt phosphorylation (Figure 5). In contrast, little is known about the function of other members of the TSC22D family in glucose metabolism.

## 6. The Role of TSC22D Family Genes in Cancer

### 6.1. TSC22D1 and Cancer

*TSC22D1* is a putative tumor suppressor that plays an important inhibitory role in malignant tumors of the salivary glands, prostate, brain, cervix uteri, and others [45,46,47,48]. Its mutations can potentially drive non-small-cell lung cancer (NSCLC) [69]. Additionally, it has an influence on the development of pulmonary adenoma in female mice [70]. However, in pancreatic ductal adenocarcinoma (PDAC), HOXA transcript at the distal tip (HOTTIP), a long non-coding RNA up-regulated in PDAC, can directly activate the *TSC22D1* gene sited on chromosome through the atypical trans-acting HOTTIP-WD repeat domain 5 (WDR5)-mixed lineage leukemia 1 (MLL1) pathway, which promotes PDAC proliferation and invasion [71]. Overall, TSC22D1 inhibits most tumors and may promote tumors in individual cases through specific pathways. In cancer, TSC-22 encoded by *TSC22D1* has also been shown to have an oncostatic effect on multiple cancers, such as salivary gland carcinomas, hematopoietic malignancies, liver tumors induced by carcinogens [25], colorectal cancers [72], and others. Its overexpression could contribute to apoptosis in cancer cells to enhance radiosensitivity and chemosensitivity [25]. As previously mentioned, TSC22D1 has two isoforms, where the short isoform of TSC22D1 has oncostatic activity, while the long isoform of TSC22D1 acts in the opposite way. Selective exhaustion of the short isoforms, or conversely, overexpression of the long isoforms, can lead to annulment of oncogene-induced senescent (OIS), which is considered as a safety mechanism to limit the outgrowth of cancer cells [1]. The TSC22D family has been extensively validated to have roles that can bind to other proteins to form large biomolecules and functions. For example, TSC22D1-2 can associate with p53 and protect it from ubiquitination-mediated degradation in cervical cancer cells [73], whereas its interaction with the intracellular tyrosine kinase domain of colony stimulator factor 1 receptor (CSF-1R) can prevent the activation of Akt and ERK pathways, thus inhibiting the transcriptional activity of NF-κB [26]. Moreover, TSC22D1 can form a complex with fibroblast growth factor receptor 2 (FGFR2) that suppresses the onset and progression of colorectal cancer (CRC) [28] (Figure 2).

### 6.2. TSC22D2 and Cancer

In addition, *TSC22D2* is also related to various cancers [6]. Nevertheless, little is known or even controversial about its biological functions and pathological mechanisms. For example, there was significantly low expression of *TSC22D2* in CRC tissues and cells compared to normal tissues, which implied poor prognosis [28]. And it was discovered that *TSC22D2* overexpression suppressed tumor cell growth, which might be partially regulated by the TSC22D2-pyruvate kinase isoform M2 (PKM2)-cyclin D1 axis. To be more specific, *TSC22D2* overexpression reduced nuclear PKM2 levels and restrained cyclin D1 expression [74]. Cyclin D1, as a key regulator of the G1/S cell cycle transition, was revealed to be a downstream gene of nuclear PKM2 [75,76]. PKM2 is a glycolytic enzyme that catalyzes the conversion of phosphopyruvate (PEP) and adenosine diphosphate (ADP) to pyruvate and adenosine triphosphate (ATP), which provides an advantage for tumor growth [77,78] and induces the expression of gene products required for tumorigenesis [79,80].

**Figure 6:**
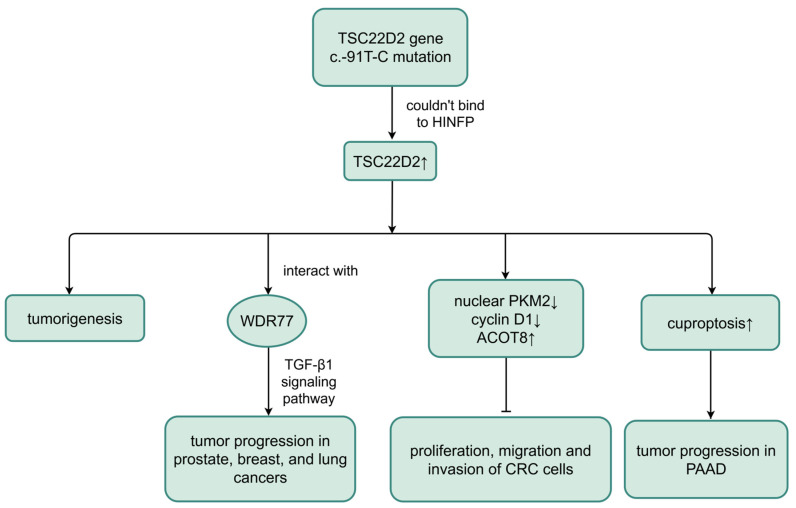
Roles of TSC22D2 in cancer. HINFP: histone H4 transcription factor; WDR77: WD repeat domain 77; TGF-β1: transforming growth factor-beta 1; PKM2: pyruvate kinase isoform M2; ACOT8: acyl-coenzyme A thioesterases 8; CRC: colorectal cancer; PAAD: pancreatic adenocarcinoma. ↑ represents up-regulated ↓ represents down-regulated, and → represents promoting ⊣ represents inhibiting. (Created in Figdraw. Wen Shen. (2026) https://www.figdraw.com).

Recently, it has been mechanistically confirmed that TSC22D2 inhibits the proliferation, migration, and invasion of CRC cells by positively regulating the expression of its downstream target acyl-coenzyme A thioesterases 8 (ACOT8) [28]. ACOT8 catalyzes the hydrolysis of lipids into fatty acids, which supply energy to tumor cells, thereby participating in the occurrence and development of various tumors [81,82]. In more detail, ACOT8 is engaged in tumor cell proliferation, survival, and metabolic reprogramming [83] and represses tumor cell apoptosis [7]. Yet another study demonstrated that *TSC22D2* was up-regulated in colorectal cancer and negatively linked to the expression of histone H4 transcription factor (HINFP) according to the GEO database (GSE4107). HINFP is a transcriptional gene regulatory inhibitor that binds to the promoter of a target gene to repress its expression. As a result of *TSC22D2* gene c.-91T-C mutation, the site where HINFP bound to the *TSC22D2* promoter region was lost, which affected the transcription and expression of *TSC22D2* and ultimately led to tumorigenesis [6]. Not singly but in pairs, *TSC22D2* was also significantly highly expressed in pancreatic adenocarcinoma (PAAD) compared to normal tissues. *TSC22D2* was identified as a cuproptosis-related gene in the univariant Cox regression, based on which researchers constructed a prognostic model for PAAD and found that *TSC22D2* was remarkably associated with overall survival of PAAD patients. Moreover, high expression of *TSC22D2* was detected as an independent predictor of poor prognosis in PAAD [8]. In cuproptosis (a new form of programmed cell death route), excess copper could bring about proteotoxic stress and cell death by binding to lipoylated protein in the tricarboxylic acid (TCA) cycle directly [84]. Copper dyshomeostasis made a great difference in cancer [85], as copper facilitated tumor proliferation, growth, angiogenesis, and metastasis [86,87]. Depletion of *TSC22D2* was found to reduce copper-induced cell death, a process called cuproptosis [8], thereby inhibiting tumor progression. However, the exact mechanism is unknown. The researchers reportedly screened for a protein WD repeat domain 77 (WDR77), which interacted with TSC22D2, through a yeast two-hybrid analysis and verified it using immunoprecipitation and immunofluorescence. WDR77 was strongly associated with a variety of tumors, including prostate, breast, and lung cancers. They hypothesized that TSC22D2 binding to WDR77 affected cell proliferation through the TGF-β1 signaling pathway and thus promoted tumor progression [30] (Figure 6). Therefore, we suggest that the expression and effect of TSC22D2 on tumors may depend on the type of tumor and TME.

### 6.3. TSC22D3 and Cancer

By interacting with various molecules or pathways, TSC22D3 affects cancer cells differently. A recent study found that P2RY8::TSC22D3 is a novel fusion associated with chemoresistance in leukemia by activating the PI3K-AKT pathway. They discovered that TSC22D3 was highly up-regulated in children with acute lymphoblastic leukemia (ALL). Such aberrant up-regulation results from promoter swapping, a genetic mechanism in which the promoter region of P2RY8 is relocated and placed in front of TSC22D3, causing TSC22D3 to be abnormally activated and overexpressed [88]. Remarkably, on one hand, it bound to and inhibited NF-κB and AP-1, thereby preventing the tumor-promoting effect. On the other hand, the induction of *TSC22D3* stimulated the proliferation of leukemia cells and ovarian cancer cells by increasing the oxidative phosphorylation (OXPHOS) of mitochondria and by activating phosphorylated Akt, respectively. Meanwhile, TSC22D3 also acted as a double-edged sword against tumors by affecting the TME, although it has not been studied thoroughly in the TME [89]. However, advances in recent research have revealed that TSC22D3 alleviated the pro-inflammatory TME by attenuating the polarization of macrophage to the M1 subtype through inhibiting the NF-κB/NOD-like receptor protein 3 (NLRP3) signaling pathway in tumor cells [90]. Furthermore, the study revealed that the expression of *TSC22D3* mRNA can be detected in tumor infiltrating immune cells, including dendritic cells (DCs), in a variety of human tumor types. Stress increased plasma corticosterone and up-regulated the expression of *TSC22D3*, which blocked type I interferon (IFN) responses in IFN-γ^+^ T cells and DC activation. High expression of *TSC22D3*, especially in DCs, disrupted cancer-therapy-induced tumor growth inhibition, and conversely, its removal from DCs eliminated glucocorticoid-mediated immunosuppression [62]. Long-GILZ (L-GILZ), a variant of the *TSC22D3* transcript, which is up-regulated by GCs, was also found to play a role in activating the p53 pathway through interaction with p53 and murine double minute 2 (MDM2) to suppress tumor growth [91] (Figure 3).

### 6.4. TSC22D4 and Cancer

Regarding the progress of *TSC22D4* in tumors, it was recently shown that *TSC22D4* is crucial for tumor proliferation and prevents cellular senescence. *TSC22D4* is expressed in normal squamous epithelium and overexpressed in squamous cell carcinomas (SCCs) [92]. TSC22D4 promoted the growth of tumor spheres in esophageal SCC cells, and knockdown of *TSC22D4* in *TSC22D4*-positive cancer cells induced cancer cell senescence [93,94]. Mechanistically, TSC22D4 is phosphorylated by the receptor tyrosine kinase pathway, which in turn enhances Ras-mediated tumorigenesis [92]. Additionally, TSC22D4 activated the IL-1-mediated inflammatory pathway through the suppression of TNF receptor-associated factor 6 (TRAF6) degradation, which mediated the continuous inflammation in tumors [95]. Another study demonstrated that hepatic TSC22D4 was associated with tumor metabolism, affecting weight loss and VLDL hyposecretion in cancer cachexia [66] (Figure 4).

## 7. TSC22D Family and Other Diseases

TSC-22 promotes the differentiation and fibrosis of myocardial fibroblasts and has a rapidly increasing expression level in response to pressure overload and myocardial injury [96]. It may regulate the expression of the cardiac collagen 3a1 gene, thereby modulating the transcription of cardiac remodeling [97]. Recently, it has also been reported that TSC22D1 can participate as a transcription factor in the regulation of Alzheimer’s disease [98], making it a possible new target for future studies.

In recent years, TSC22D2 has begun to be studied in more than tumor-related settings. In aspects of immunology, anti-inflammatory miRNAs in macrophage-derived extracellular vesicles (MDEVs) directly lowered the expression of *TSC22D2*, up-regulated the differentiation of Tregs through the TSC22D2-signal transducer and activator of transcription 3 (STAT3) pathway, and suppressed m1-macrophage polarization via the TSC22D2-adenosine monophosphate-activated protein kinase α (AMPKα)/mammalian target of rapamycin protein (mTOR) pathway, thereby affecting the immunoregulatory network [99].

In addition, researchers identified *TSC22D2* as one of the differentially expressed genes with the highest degree scores specific to severe acute respiratory syndrome (SARS) in the immunoregulatory network by performing a topological analysis of hub-bottleneck genes of union motif networks in vitro, and functional enrichment analysis suggested that it might be involved in SARS-associated immunomodulation by regulating the macromolecule metabolic process [100]. In the cardiovascular system, the sequential expression of the differentially expressed gene *TSC22D2* was described to remain up-regulated after acute myocardial infarction (AMI), indicating that it plays an important role in the development of AMI [101]. Furthermore, differentially expressed proteins between polycystic ovary syndrome-insulin resistance (PCOS-ir), PCOS-non-ir, and control groups were identified and analyzed to confirm that TSC22D2 was a key protein affecting live birth in patients with PCOS [102].

TSC22D3 was also found to work in the liver. Decreased expression of GR and TSC22D3 in liver Kupffer cells and increased release of hepatic pro-inflammatory cytokines resulted in an inflammatory state in the livers of obese mice [103]. In addition, hepatic TSC22D3 levels were lower in patients with liver fibrosis, and *TSC22D3* deficiency promoted the development of liver fibrosis. This might be related to the inhibition of hepatic macrophages and CD4^+^ T-cell recruitment when TSC22D3 downregulated C-C motif chemokine ligand 2 (CCL2) expression [104]. Additionally, the study revealed that the glucocorticoid system and *TSC22D3* are clinically relevant in sepsis and the C-allele of rs3747406 poses a risk for sepsis mortality for sequential organ failure assessment (SOFA) scores higher than 6, implying that *TSC22D3* gene polymorphisms contribute to variance in sepsis mortality rates [105]. More interestingly, TSC22D3 could promote hair follicle regeneration and stem cell activation by enhancing FOXP3 and GR expression within the hair follicle immune-ecological niche, which is an emerging potential target for treating hair loss [106].

## 8. Conclusions

Together, TSC22D family genes inhibit or promote cancer depending on the tumor microenvironment or different tumor types. In terms of metabolic regulation, TSC22D1 controls systemic HDL cholesterol metabolism. The fact that the WC-related SNP near the TSC22D2 gene is linked to circulating lipocalin levels indicates an association between TSC22D2 and abdominal obesity. TSC22D3 inhibits key regulators of adipogenesis and blocks adipocyte differentiation. TSC22D4 regulates serum TG homeostasis via the control of hepatic VLDL release and modulates insulin sensitivity and gluconeogenesis through the effect on Akt phosphorylation. In summary, TSC22D family genes represent a critical nexus in the regulation of metabolism and cancer biology. Its complex roles and regulatory networks offer valuable insights into disease pathogenesis and open promising pathways for innovative clinical interventions. Continued interdisciplinary research efforts will be essential to translate these scientific advances into tangible benefits for patients suffering from metabolic diseases and cancer.

## Data Availability

No new data were created or analyzed in this study.
